# Effects of *Artemisia ordosica Krasch*. aqueous extract on antioxidant and immune functions of broilers

**DOI:** 10.3389/fvets.2026.1857679

**Published:** 2026-06-26

**Authors:** Gen Gang, Manman Tong, Xiaoyu Guo, Yanli Zhao, Yongmei Guo, Qingyue Zhang, Binlin Shi, Sumei Yan

**Affiliations:** College of Animal Science, Inner Mongolia Agricultural University, Key Laboratory of Animal Nutrition and Feed Science at Universities of Inner Mongolia Autonomous Region, Hohhot, China

**Keywords:** antioxidative index, *Artemisia ordosica Krasch*. aqueous extract, broiler, immune function, small intestine

## Abstract

**Introduction:**

This study investigated the effects of *Artemisia ordosica Krasch*. aqueous extract (AOE) on the antioxidant and immune functions of broilers. Intensive broiler production often faces challenges from oxidative stress and compromised immunity, prompting a search for effective, green-feed additives.

**Methods:**

A total of 160 fifteen-day-old Arbor Acre broilers were randomly allocated to five dietary treatment groups, receiving a basal diet supplemented with AOE at 0, 250, 500, 1000, or 2000 mg/kg for 28 days. Serum and intestinal (duodenum, jejunum, ileum) samples were collected at 28 and 42 days of age for analysis.

**Results:**

The results showed that dietary AOE supplementation could significantly improve the antioxidant capacity of broilers: at 42 days of age, it increased serum CAT activity and decreased MDA concentration; it also enhanced the activity of GSH-Px, SOD, CAT and T-AOC in the intestine, and reduced ileal MDA content. In terms of immune function, AOE increased serum IL-2, IL-1β, IL-6, TNF-α levels at 28 days of age and Newcastle disease antibody titers at both 28 and 42 days of age. At the molecular level, AOE upregulated the mRNA expression of CAT, SOD, GSH-Px in the duodenum and ileum at 28 days of age and increased the mRNA expression of iNOS in the intestine, consistent with the elevated serum NO content and iNOS activity. The regulatory effects of AOE were more significant at 42 days of age, and the appropriate supplementation level was 1000 mg/kg.

**Conclusion:**

In conclusion, AOE can be used as a natural green feed additive to enhance the antioxidant capacity and immune function of broilers, providing experimental basis for its application in broiler production.

## 1 Introduction

Modern poultry production is centered on intensive farming systems, where factors such as high stocking density, environmental fluctuations, and rapid metabolism readily lead to oxidative stress imbalance and immune dysfunction in broilers (1). Oxidative stress results from an imbalance between ROS production and the scavenging capacity of the body's antioxidant defense system (2). This imbalance triggers cellular damage and physiological dysfunction, significantly increasing disease susceptibility in broilers and subsequently affecting production efficiency (3). Therefore, developing safe and effective green feed additives to alleviate oxidative stress and strengthen immune defense has become an important research direction in intensive broiler production (4, 5). Natural plant extracts, rich in bioactive compounds such as polyphenols, flavonoids, polysaccharides, and essential oils, have emerged as a core research category for green feed additives (6). These compounds possess well-documented antioxidant and immunomodulatory activity (7, 8). Numerous studies have confirmed that dietary supplementation with plant-derived products can effectively improve the antioxidant status and enhance immune function in poultry, providing a feasible approach for addressing health challenges in intensive farming systems (9–11).

*Artemisia ordosica Krasch*. (*A. ordosica*) is a perennial shrub of the Asteraceae family, widely distributed in the arid and semi-arid regions of northwest China (12, 13). As a traditional medicinal plant, it is rich in flavonoids, polysaccharides, volatile oils, and phenolic acids, all of which possess distinct biological activity (14–16). In broilers, *A. ordosica* polysaccharides can upregulate the gene expression of NRF2 and HO-1 in intestinal tissues via the NRF2/HO-1 pathway, while simultaneously activating the TLR4/NF-κB/MAPK pathway to regulate inflammatory cytokines (17, 18). *In vitro* experiments have also demonstrated that *A. ordosica* polysaccharides exhibit concentration-dependent DPPH, hydroxyl radical, and ABTS radical scavenging capacity as well as reducing power (19). *A. ordosica* flavonoids can alleviate LPS-induced decreases in serum antioxidant enzyme activity in broilers, reduce oxidative damage markers such as ROS and 8-hydroxy2′-deoxyguanosine (8-OHdG), as well as tissue malondialdehyde (MDA) content, and mitigate the negative impact of inflammatory stress on immune function by activating the NRF2 pathway (20). Studies in rat models have also confirmed their ability to enhance serum and liver antioxidant enzyme activity and T-AOC, up-regulate SOD1, SOD2, and CAT gene expression, and reduce MDA content (19). Li et al. (21), using lactating donkeys as research subjects, found that dietary supplementation with crude *A. ordosica* polysaccharides significantly elevated serum SOD and CAT activity and T-AOC, decreased MDA and ROS levels, and effectively alleviated oxidative stress during lactation. Concurrently, it upregulated serum immunoglobulin A (IgA), IgG, and IgM concentration, achieving synergistic enhancement of antioxidant and immune functions. However, due to its simple preparation process, the aqueous extract of *A. ordosica* (AOE) contains quantifiable key bioactive components including total polysaccharides, total phenolics, and total flavonoids, with *in vitro* DPPH radical scavenging activity reaching 85.52 ± 0.10%, demonstrating a clear antioxidant material basis. Further studies using weaned piglets as research subjects have revealed its serum antioxidant and immunoregulatory potential (22).

Nevertheless, significant research gaps remain: the antioxidant and immunomodulatory effects of AOE in broiler chickens have not yet been elucidated. The intestine serves as a critical site for nutrient absorption and constitutes the core barrier of mucosal immune defense (23). Clarifying the synergistic regulation between local intestinal and systemic responses is of great significance for elucidating the overall mechanism of action of AOE. Furthermore, NO and iNOS, as key molecules in immune regulation and oxidative stress balance, have not been investigated regarding their roles in AOE-mediated regulation of broiler health. Therefore, this study aims to test the following hypothesis: dietary supplementation with AOE can enhance the antioxidant and immune functions of broilers by regulating the expression of antioxidant-related genes and iNOS molecules. The core objective of this research is to elucidate the modulatory effects of different dietary levels of AOE on serum and intestinal segment antioxidant and immune parameters in broilers, determine the appropriate dietary supplementation level of AOE, and provide scientific evidence for its application as a green feed additive in broiler production.

## 2 Methods

### 2.1 Preparation of *Artemisia ordosica Krasch*. aqueous extract

Whole *A. ordosica* plants were collected from the sandy grassland in Ordos, Inner Mongolia, China, during the flowering period (August). The plants were air-dried naturally, then ground into a coarse powder using a pulverizer. The powder was mixed with water (1:10, w/v) and boiled for 2 h. The extraction process was repeated twice. The combined aqueous extracts were filtered through cheesecloth and centrifuged at 4,000 × g for 15 min to remove insoluble particles. The supernatant was concentrated under reduced pressure using a rotary evaporator at 60 °C and freeze-dried to obtain AOE powder (22). The final product was stored at −20 °C until used in diet formulation.

### 2.2 Animals, diets, and experimental design

A total of 160 one-day-old Arbor Acre broiler chicks (half male and half female) were obtained from a commercial hatchery. The chicks were raised on a plastic mesh floor in the experimental poultry house. They were fed a standard corn-soybean meal basal diet for the first 14 days. At 15 days of age, the chicks were weighed and randomly allocated to five dietary treatment groups with 4 replicates per group and 8 birds per replicate (4 males and 4 females per replicate). The initial body weight did not differ significantly among groups (*P* > 0.05). The five dietary treatments included a basal diet (Control, 0 mg/kg AOE) and the basal diet supplemented with AOE at 250, 500, 1,000, or 2,000 mg/kg, respectively. The basal diet was formulated according to Agricultural Industry Standard of the People's Republic of China - Standard for Chicken Rearing (NY/T 33-2004) for broilers (24), with the composition and nutrient levels shown in Table 1. The experimental period lasted for 28 days (15~42 days of age), divided into the early stage (15~28 days of age) and late stage (29~42 days of age). All birds had *ad libitum* access to feed and water throughout the experiment, and other feeding procedures followed conventional standards. Routine immunization was performed as follows: Newcastle disease (ND) vaccine was administered via nasal and ocular drops at 1 day of age; ND + infectious bronchitis (IB) attenuated vaccine was given via nasal and ocular drops at 7 days of age; and bursal disease vaccine was provided via drinking water at 14 days of age.

**Table 1:** Composition and nutrient levels of basal diets (air-dry basis) %.

Items	Content	
	15~28 days of age	29~42 days of age
Ingredients		
Corn	51.68	58.49
Soybean meal	41.00	34.30
Soybean oil	3.00	3.00
Dicalcium phosphate	1.90	1.80
Limestone	1.10	1.20
Salt	0.37	0.37
98% Lysine	0.05	0.03
Methionine	0.19	0.10
Premix ^1^	0.71	0.71
Total	100.00	100.00
Nutrient levels ^2^		
ME (MJ/kg)	12.62	12.87
CP	21.84	19.95
Ca	1.00	1.00
AP	0.48	0.46
Lys	1.40	1.20
Met	0.56	0.44
L-Cys	0.40	0.37

^1^The premix provided the following nutrient content for one kilogram of diet: vitamin A 6141.5 IU, vitamin D3 1789.2 IU, vitamin E 7.99 mg, vitamin K 1.82 mg, vitamin B1 0.65 mg, vitamin B_2_ 3.93 mg, vitamin B6 2.08 mg, vitamin B1_2_ 0.01 mg, nicotinic acid 18.06 mg, calcium pantothenate 6.65 mg, folic acid 0.59 mg, biotin 0.07 mg, choline chloride 332.28 mg, Fe 60.91 mg, Cu 6.01 mg, Zn 65.75 mg, Mn 62.3 mg, I 0.9 mg, Se 0.21 mg.

^2^ME: Metabolizable energy, CP: Crude protein, Ca: Calcium, AP: Available phosphorus, Lys: Lysine, Met: Methionine, L-Cys: L-Cystine. Metabolizable energy was a calculated value, while the other nutrient levels were measured values.

### 2.3 Sample collection

At 28 and 42 days of age, 2 birds per replicate (one male and one female, 8 birds per treatment group) were sampled after 12 h of fasting. Blood samples were collected from the wing vein into non-heparinized tubes. The samples were allowed to clot at room temperature for 1 h and then centrifuged at 1200 × g for 15 min at 4 °C to obtain serum, which was stored at −80 °C for subsequent analyses. Following blood collection, the birds were euthanized by cervical dislocation. The abdominal cavity was opened, and the small intestine was excised. Segments of approximately 2 cm from the middle of the duodenum, jejunum, and ileum were collected, immediately snap-frozen in liquid nitrogen, and stored at −80 °C for gene expression analysis. Another set of intestinal tissue samples were wrapped in tin foil, stored at −20°C, and homogenized with 9 volumes of normal saline on ice before centrifugation at 1200 × g for 10 min. The supernatant was collected for antioxidant enzyme activity determination.

### 2.4 Determination of antioxidant and immune indices

The activity of SOD, glutathione peroxidase (GSH-Px), CAT, and T-AOC, the concentration of MDA, as well as NO content and iNOS activity, were measured using commercial kits according to the manufacturer's instructions (Jiancheng Bioengineering Institute, Nanjing, China). The concentrations of cytokines, including IL-1β, IL-2, IL-4, IL-6, IFN-γ, and TNF-α, were determined using chicken-specific ELISA kits in line with the manufacturer's guidelines (Wuhan Colorful Gene Biological Technology Co., LTD, Wuhan, China). Specific antibody titers against NDV were measured by hemagglutination inhibition test using 96-well “U”-shaped microtiter plates.

### 2.5 RT-qPCR analysis

Total RNA was extracted from frozen intestinal tissue samples (100 mg) using RNAiso™ Plus reagent (Accurate Biotechnology Co. Ltd, Changsha, China). The integrity of RNA was verified by 1.5% agarose gel electrophoresis, and purity (OD260/OD280 = 1.8~2.2) and concentration were measured using a microplate reader. First-strand cDNA was synthesized using the PrimeScript™ RT Kit (Table 2). The primer sequences for target genes (GSH-Px, CAT, SOD, iNOS) and the reference gene (β-actin) were designed based on GenBank sequences and synthesized by BGI Genomics Co., Ltd. RT-qPCR was conducted on a CFX96 Real-Time PCR Detection System (Bio-Rad, USA) with SYBR^®^ Premix Ex Taq™ II (TaKaRa Biotechnology Co. Ltd., Dalian, China). The reaction was performed under the following conditions: 95 °C for 30 s, followed by 40 cycles of 95 °C for 5 s, 55 °C for 20 s, and 72 °C for 20 s. Gene expression levels were analyzed using the 2^−Δ*ΔCt*^ method.

**Table 2:** Primer sequences and parameters.

Genes^1^	GenBank accession No.	Primer sequences (5^′^3^′^)^2^	Length/bp
*β-actin*	NM_205518	F: GCCAACAGAGAGAAGATGACAC	118
		R: GTAACACCATCACCAGAGTCCA	
*SOD*	NM_205064.1	F: TTGTCTGATGGAGATCATGGCTTC	98
		R: TGCTTGCCTTCAGGATTAAAGTGA	
*CAT*	NM_001031215.1	F: GTTGGCGGTAGGAGTCTGGTCT	182
		R: GTGGTCAAGGCATCTGGCTTCTG	
*GSH-Px*	NM_001163245.1	F: CAAAGTTGCGGTCAGTGGA	136
		R: AGAGTCCCAGGCCTTTACTACTTTC	
*iNOS*	NM_204961.1	F: GCAGCACGTGGCTGAACAA	165
		R: CATAGAGACGCTGCTGCCAGA	

^1^β-actin: beta-actin; SOD: Superoxide dismutase; CAT: Catalase; GSH-Px: Glutathione peroxidase; iNOS: Inducible nitric oxide synthase.

^2^F: forward primer; *R*: reverse primer.

### 2.6 Statistical analysis

All data were organized using Microsoft Excel 2007 and analyzed with SAS 9.0 software (SAS Institute Inc., USA). Orthogonal polynomial contrasts were performed to determine the linear and quadratic effects of increasing dietary WEAA levels on the measured indices, thereby determining the dose-dependent effects of each index on AOE. *P* < 0.05 was considered statistically significant, and *P* < 0.01 was extremely significant. The standard error of the mean (SEM) was reported for all data.

## 3 Results

### 3.1 Effects of AOE on serum-related indices in broilers

The effects of dietary AOE supplementation on serum antioxidant indices in broilers are presented in Table 3. At 28 days of age, dietary AOE supplementation had no significant effect on serum antioxidant indices. At 42 days of age, serum CAT activity increased quadratically (*P* = 0.012), whereas serum MDA concentration decreased linearly or quadratically with increasing AOE levels (*P* = 0.032 and *P* = 0.040, respectively). There was no significant difference in GSH-Px, SOD activity, or T-AOC (*P* > 0.05).

**Table 3:** Effects of dietary AOE on antioxidant indices in serum of broilers.

Item^1^	AOE supplemental level, mg/kg^2^					SEM^3^	*P*-value^4^	
	0	250	500	1,000	2,000		Linear	Quadratic
GSH-Px/(pg/mL)								
28 d	21.21	33.52	29.95	22.67	21.29	2.90	0.412	0.388
42 d	44.99	41.71	38.43	42.89	38.63	2.45	0.370	0.669
CAT/(pg/mL)								
28 d	32.36	48.73	55.98	45.70	42.73	5.30	0.796	0.171
42 d	21.07	31.95	34.55	31.68	28.07	2.25	0.562	^*^0.012
SOD/(ng/mL)								
28 d	36.52	49.46	51.42	44.42	42.39	2.89	0.749	0.301
42 d	81.92	88.87	93.24	87.12	86.60	7.20	0.925	0.824
T-AOC/(U/mL)								
28 d	4.99	6.11	5.67	5.08	5.95	0.36	0.555	0.740
42 d	2.81	3.71	3.97	3.96	3.10	0.38	0.968	0.064
MDA/(mmol/mL)								
28 d	17.76	17.48	15.41	16.15	16.55	1.07	0.607	0.556
42 d	16.25	15.30	13.25	11.92	14.09	1.20	^*^0.032	^*^0.040

^1^T-AOC: Total antioxidant capacity; SOD: Superoxide dismutase; CAT: Catalase; GSH-Px: Glutathione peroxidase; MDA: Malondialdehyde.

^2^AOE: *Artemisia ordosica Krasch*. aqueous extract.

^3^SEM: Standard error of the mean.

^4^Dose-dependent effects of AOE. ^*^P ≤ 0.05 indicates a significant dose-dependent effect; ^**^P ≤ 0.01 indicates an extremely significant dose-dependent effect.

The effects of dietary AOE supplementation on serum cytokine levels in broilers are presented in Table 4. At 28 days of age, as dietary AOE levels increased, serum IL-2, IL-1β, IL-6, and TNF-α exhibited quadratic increase (*P* = 0.015, *P* = 0.017, *P* = 0.012, *P* = 0.015, respectively). At 42 days of age, with increasing AOE levels, serum IL-1β (*P* = 0.023) and IL-6 (*P* = 0.022) increased quadratically, and TNF-α increased linearly or quadratically (*P* = 0.053; *P* = 0.015).

**Table 4:** Effects of dietary AOE on the level of cytokines in serum of broilers (pg/mL).

Item^1^	AOE supplemental level, mg/kg^2^					SEM^3^	*P*-value^4^	
	0	250	500	1,000	2,000		Linear	Quadratic
IL-2								
28 d	261.8	289.5	284.1	316.9	282.8	11.08	0.421	^*^0.015
42 d	273.5	273.2	271.5	273.7	275.6	9.72	0.868	0.978
IL-4								
28 d	87.39	86.55	87.83	88.57	86.78	1.39	0.991	0.804
42 d	81.52	79.40	81.52	81.88	82.93	1.50	0.258	0.533
IL-1β								
28 d	464.6	471.2	490.0	496.6	480.5	7.27	0.273	0.017
42 d	355.1	390.4	415.4	461.1	425.3	22.46	0.075	^*^0.023
IL-6								
28 d	30.07	31.11	32.94	33.02	30.50	0.74	0.989	^*^0.012
42 d	30.82	31.44	31.38	34.56	32.36	0.76	0.126	^*^0.022
IFN-γ								
28 d	79.51	82.17	83.98	84.73	79.37	2.23	0.767	0.121
42 d	85.45	84.72	84.19	95.01	84.05	2.35	0.961	0.104
TNF-α								
28 d	79.47	81.11	83.4	85.74	81.45	1.34	0.505	^*^0.015
42 d	78.60	80.18	83.16	85.02	83.53	1.43	0.053	^*^0.015

^1^IL-1β: Interleukin-1β; IL-2: Interleukin-2; IL-4: Interleukin-4; IL-6: Interleukin-6; IFN-γ: Interferon-γ; TNF-α: Tumor necrosis factor -α.

^2^AOE: *Artemisia ordosica Krasch*. aqueous extract.

^3^SEM: Standard error of the mean.

^4^Dose-dependent effects of AOE. ^*^P ≤ 0.05 indicates a significant dose-dependent effect; ^**^P ≤ 0.01 indicates an extremely significant dose-dependent effect.

The effects of dietary AOE supplementation on serum Newcastle disease anti-body titers in broilers are presented in Table 5. At 28 days of age, with increasing AOE levels, serum antibody titers increased linearly and quadratically (*P* < 0.001; *P* < 0.001). At 42 days of age, serum antibody titers increased linearly (*P* = 0.031).

**Table 5:** Effects of dietary AOE on Newcastle disease antibody titer in serum of broilers (Log2X).

Item	AOE supplemental level, mg/kg^**1**^					SEM^2^	***P***-value^**3**^	
	0	250	500	1,000	2,000		Linear	Quadratic
28 d	3.63	4.63	5.13	6.25	6.50	0.50	^**^ < 0.001	^**^ < 0.001
42 d	4.33	4.71	4.67	5.00	5.60	0.36	^*^0.031	0.101

^1^AOE: *Artemisia ordosica Krasch*. aqueous extract.

^2^SEM: Standard error of the mean.

^3^Dose-dependent effects of AOE. ^*^P ≤ 0.05 indicates a significant dose-dependent effect; ^**^P ≤ 0.01 indicates an extremely significant dose-dependent effect.

The effects of dietary AOE supplementation on serum NO content and iNOS activity in broilers are presented in Table 6. At 28 days of age, as dietary AOE levels increased, serum NO content exhibited significant linear or quadratic increase (*P* = 0.011, *P* = 0.004), whereas serum iNOS activity increased linearly (*P* = 0.051). At 42 days of age, with increasing AOE levels, serum NO content increased linearly (*P* = 0.039), whereas serum iNOS activity increased linearly or quadratically (*P* = 0.029; *P* = 0.041).

**Table 6:** Effects of dietary AOE on NO content and iNOS activity in serum of broilers.

Item^1^	AOE supplemental level, mg/kg^2^					SEM^3^	*P*-value^4^	
	0	250	500	1,000	2,000		Linear	Quadratic
NO (μmol/L)								
28 d	3.62	5.24	4.28	6.07	5.52	0.44	^*^0.011	^**^0.004
42 d	6.21	6.55	8.21	7.09	8.36	0.52	^*^0.039	0.114
iNOS (U/mL)								
28 d	10.83	16.51	15.13	11.9	17.22	1.38	0.051	0.155
42 d	12.33	14.38	15.09	16.52	16.77	1.17	^*^0.029	^*^0.041

^1^NO: Nitric oxide; iNOS: Inducible nitric oxide synthase.

^2^AOE: *Artemisia ordosica Krasch*. aqueous extract.

^3^SEM: Standard error of the mean.

^4^Dose-dependent effects of AOE. ^*^P ≤ 0.05 indicates a significant dose-dependent effect; ^**^P ≤ 0.01 indicates an extremely significant dose-dependent effect.

### 3.2 Effects of AOE on intestinal-related indices in broilers

The effects of dietary AOE supplementation on antioxidant indices in the duodenum of broilers are presented in Table 7. At 28 days of age, duodenal SOD activity and T-AOC increased quadratically (*P* = 0.016; *P* = 0.038) with increasing AOE levels. At 42 days of age, duodenal CAT, SOD activity and T-AOC increased quadratically (*P* = 0.038; *P* = 0.046 and *P* = 0.046, respectively) with increasing AOE levels.

**Table 7:** Effects of dietary AOE on antioxidant indices in duodenum of broilers.

Item^1^	AOE supplemental level, mg/kg^2^					SEM^3^	*P*-value^4^	
	0	250	500	1,000	2,000		Linear	Quadratic
GSH-Px/(U/mg prot.)								
28 d	7.11	8.34	8.54	8.08	7.73	0.45	0.866	0.362
42 d	5.51	6.79	8.81	7.24	6.67	0.61	0.991	0.095
CAT/(U/mg prot.)								
28 d	1.52	1.61	1.54	1.50	1.54	0.28	0.962	0.998
42 d	0.44	0.63	1.07	0.76	0.84	0.10	0.080	^*^0.038
SOD/(U/mg prot.)								
28 d	108.01	128.83	124.24	135.59	119.21	5.23	0.714	^*^0.016
42 d	211.77	256.51	370.65	258.89	243.95	21.72	0.731	^*^0.046
T-AOC/(U/mg prot.)								
28 d	1.05	1.01	1.27	1.34	1.25	0.09	0.067	^*^0.038
42 d	1.01	1.16	1.14	1.18	0.91	0.08	0.168	^*^0.046
MDA/(nmol/mg prot.)								
28 d	0.83	0.77	0.69	0.77	0.79	0.07	0.980	0.787
42 d	1.00	0.95	0.91	0.78	0.86	0.10	0.323	0.368

^1^T-AOC: Total antioxidant capacity; SOD: Superoxide dismutase; CAT: Catalase; GSH-Px: Glutathione peroxidase; MDA: Malondialdehyde.

^2^AOE: *Artemisia ordosica Krasch*. aqueous extract.

^3^SEM: Standard error of the mean.

^4^Dose-dependent effects of AOE. ^*^P ≤ 0.05 indicates a significant dose-dependent effect; ^**^P ≤ 0.01 indicates an extremely significant dose-dependent effect.

The effects of dietary AOE supplementation on antioxidant indices in the jejunum of broilers are presented in Table 8. At 28 days of age, dietary AOE supplementation had no significant effect. At 42 days of age, with increasing AOE levels, jejunal GSH-Px activity increased quadratically (*P* = 0.013), and SOD activity increased linearly or quadratically (*P* = 0.051; *P* = 0.022).

**Table 8:** Effects of dietary AOE on antioxidant indices in jejunum of broilers.

Item^1^	AOE supplemental level, mg/kg^2^					SEM^3^	*P*-value^4^	
	0	250	500	1,000	2,000		Linear	Quadratic
GSH-Px/(U/mg prot.)								
28 d	11.44	15.10	20.86	17.84	17.73	2.12	0.295	0.139
42 d	9.95	12.83	13.80	14.49	9.71	1.25	0.433	^*^0.013
CAT/(U/mg prot.)								
28 d	3.85	4.30	3.54	4.05	4.11	0.26	0.726	0.937
42 d	2.86	2.81	2.66	3.37	2.72	0.35	0.992	0.763
SOD/(U/mg prot.)								
28 d	164.53	172.90	166.75	182.15	206.26	18.24	0.096	0.248
42 d	215.42	225.73	237.99	250.58	242.60	9.11	0.051	^*^0.022
T-AOC/(U/mg prot.)								
28 d	2.79	2.54	2.62	2.77	3.47	0.37	0.116	0.221
42 d	0.85	0.89	0.86	0.95	0.84	0.04	0.935	0.450
MDA/(nmol/mg prot.)								
28 d	1.54	1.42	1.58	1.39	1.33	0.14	0.438	0.744
42 d	0.96	1.00	0.62	1.02	0.81	0.10	0.560	0.837

^1^T-AOC: Total antioxidant capacity; SOD: Superoxide dismutase; CAT: Catalase; GSH-Px: Glutathione peroxidase; MDA: Malondialdehyde.

^2^AOE: *Artemisia ordosica Krasch*. aqueous extract.

^3^SEM: Standard error of the mean.

^4^Dose-dependent effects of AOE. ^*^P ≤ 0.05 indicates a significant dose-dependent effect; ^**^P ≤ 0.01 indicates an extremely significant dose-dependent effect.

The effects of dietary AOE supplementation on antioxidant indices in the ileum of broilers are presented in Table 9. At 28 days of age, ileal GSH-Px activity exhibited significant quadratic increase (*P* = 0.006), whereas T-AOC increased linearly or quadratically (*P* < 0.001; *P* < 0.001) with increasing AOE levels. At 42 days of age, with increasing AOE levels, ileal GSH-Px, CAT, SOD activity increased quadratically (*P* = 0.030; *P* = 0.036; *P* = 0.032), and T-AOC increased linearly or quadratically (*P* = 0.049; *P* = 0.049), whereas MDA content decreased linearly (*P* = 0.024).

**Table 9:** Effects of dietary AOE on antioxidant indices in ileum of broilers.

Item^1^	AOE supplemental level, mg/kg^2^					SEM^3^	*P*-value^4^	
	0	250	500	1,000	2,000		Linear	Quadratic
GSH-Px/(U/mg prot.)								
28 d	11.17	13.75	17.96	21.59	17.99	2.11	0.068	^**^0.006
42 d	12.44	15.90	17.53	18.65	17.39	1.58	0.105	^*^0.030
CAT/(U/mg prot.)								
28 d	1.55	1.71	1.68	1.81	1.99	0.18	0.164	0.385
42 d	2.27	2.08	2.46	3.13	2.66	0.21	0.072	^*^0.036
SOD/(U/mg prot.)								
28 d	105.33	100.13	104.82	100.47	106.50	7.39	0.903	0.738
42 d	201.95	210.11	209.74	236.11	209.55	8.29	0.451	^*^0.032
T-AOC/(U/mg prot.)								
28 d	1.10	1.34	2.17	2.30	2.54	0.26	^**^ < 0.001	^**^ < 0.001
42 d	0.90	0.81	0.92	1.13	1.03	0.08	^*^0.045	^*^0.049
MDA/(nmol/mg prot.)								
28 d	2.59	1.32	1.78	1.79	2.00	0.22	0.811	0.180
42 d	1.19	1.05	1.06	0.92	0.77	0.12	^*^0.024	0.078

^1^T-AOC: Total antioxidant capacity; SOD: Superoxide dismutase; CAT: Catalase; GSH-Px: Glutathione peroxidase; MDA: Malondialdehyde.

^2^AOE: *Artemisia ordosica Krasch*. aqueous extract.

^3^SEM: Standard error of the mean.

^4^Dose-dependent effects of AOE. ^*^P ≤ 0.05 indicates a significant dose-dependent effect; ^**^P ≤ 0.01 indicates an extremely significant dose-dependent effect.

### 3.3 Effects of AOE on intestinal antioxidant related gene expression in broilers

**Figure 1:**
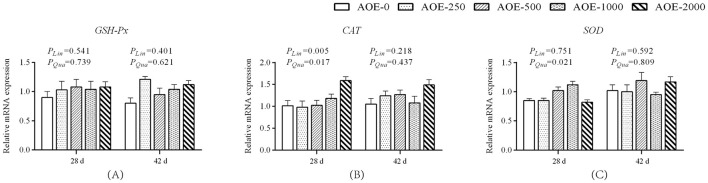
Effects of dietary AOE supplementation on antioxidant gene mRNA expression in the duodenum of broilers. **(A)** GSH-Px: Glutathione peroxidase, **(B)** CAT: Catalase, **(C)** SOD: Superoxide dismutase. AOE: *Artemisia ordosica Krasch*. aqueous extract. CON: control; AOE-250: 250 mg/kg AOE; AOE-500: 500 mg/kg AOE; AOE-1000: 1,000 mg/kg AOE; AOE-1500: 1,500 mg/kg AOE; AOE-2000: 2,000 mg/kg AOE.

The effects of dietary AOE supplementation on antioxidant related gene expression in the duodenum of broilers are presented in Figure 1. At 28 days of age, as dietary AOE levels increased, duodenal *CAT* mRNA expression exhibited significant linear or quadratic increase (*P* = 0.005; *P* = 0.017), whereas *SOD* mRNA expression increased quadratically (*P* = 0.021). At 42 days of age, dietary AOE supplementation had no significant effect on the antioxidant related gene expression in the duodenum.

**Figure 2:**
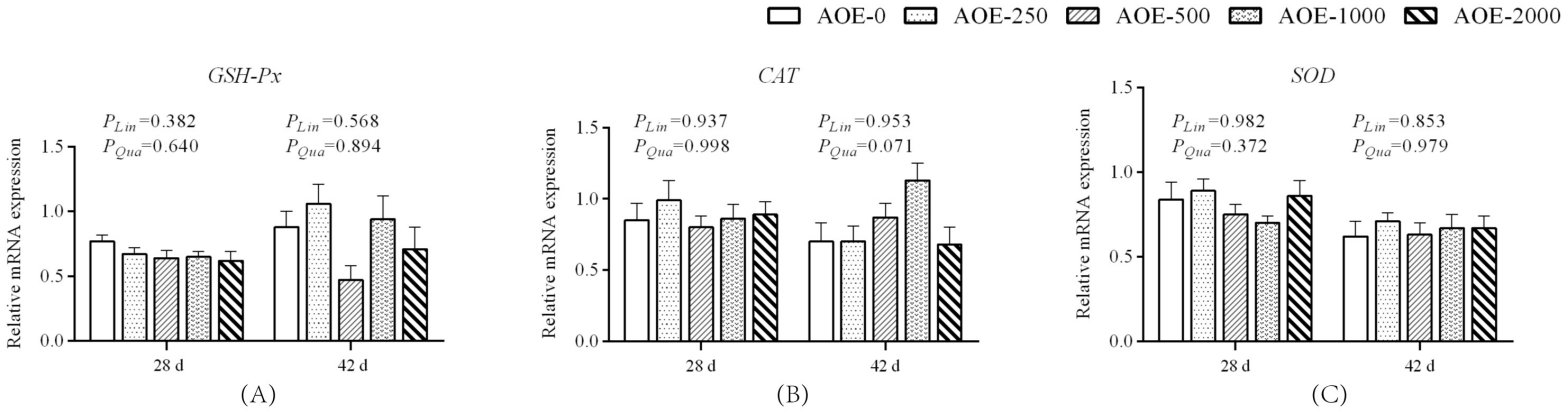
Effects of dietary AOE supplementation on antioxidant related gene expression in the jejunum of broilers. **(A)** GSH-Px: Glutathione peroxidase, **(B)** CAT: Catalase, **(C)** SOD: Superoxide dismutase. AOE: *Artemisia ordosica Krasch*. aqueous extract. CON: control; AOE-250: 250 mg/kg AOE; AOE-500: 500 mg/kg AOE; AOE-1000: 1,000 mg/kg AOE; AOE-1500: 1,500 mg/kg AOE; AOE-2000: 2,000 mg/kg AOE.

The effects of dietary AOE supplementation on antioxidant related gene expression in the jejunum of broilers are presented in Figure 2. Dietary AOE supplementation had no significant effect on the antioxidant related gene expression in the jejunum.

**Figure 3:**
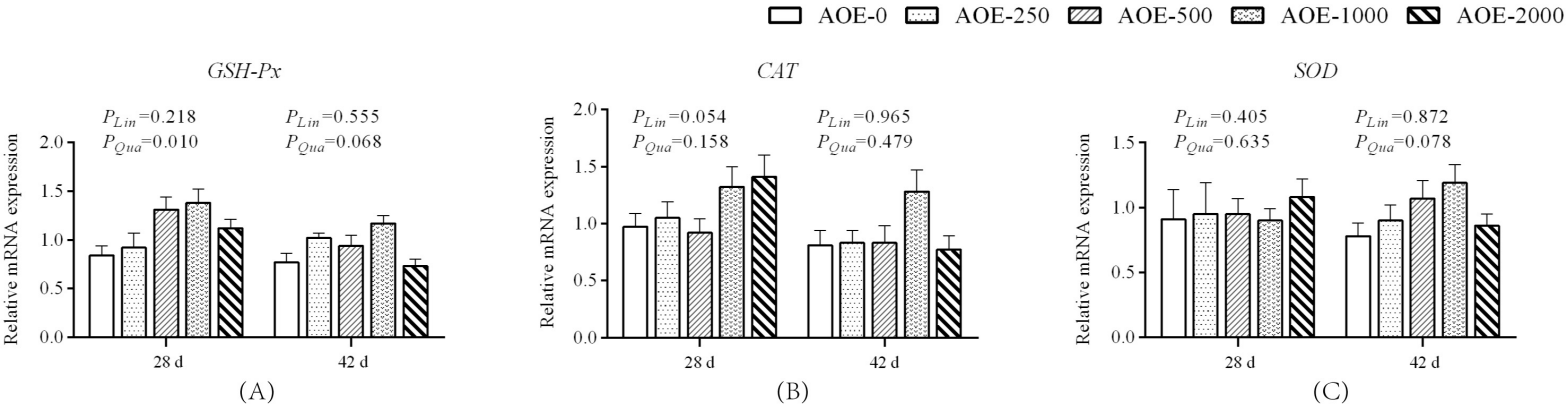
Effects of dietary AOE supplementation on antioxidant gene mRNA expression in the ileum of broilers. **(A)** GSH-Px: Glutathione peroxidase, **(B)** CAT: Catalase, **(C)** SOD: Superoxide dismutase. AOE: *Artemisia ordosica Krasch*. aqueous extract. CON: control; AOE-250: 250 mg/kg AOE; AOE-500: 500 mg/kg AOE; AOE-1000: 1,000 mg/kg AOE; AOE-1500: 1,500 mg/kg AOE; AOE-2000: 2,000 mg/kg AOE.

The effects of dietary AOE supplementation on antioxidant related gene expression in the ileum of broilers are presented in Figure 3. At 28 days of age, as dietary AOE levels increased, ileal *GSH-Px* mRNA expression exhibited significant quadratic increase (*P* = 0.010), whereas *CAT* mRNA expression increase linearly (*P* = 0.054). At 42 days of age, dietary AOE supplementation had no significant effect on the antioxidant related gene expression in the ileum.

### 3.4 Effects of AOE on intestinal *iNOS* mRNA expression in broilers

**Figure 4:**
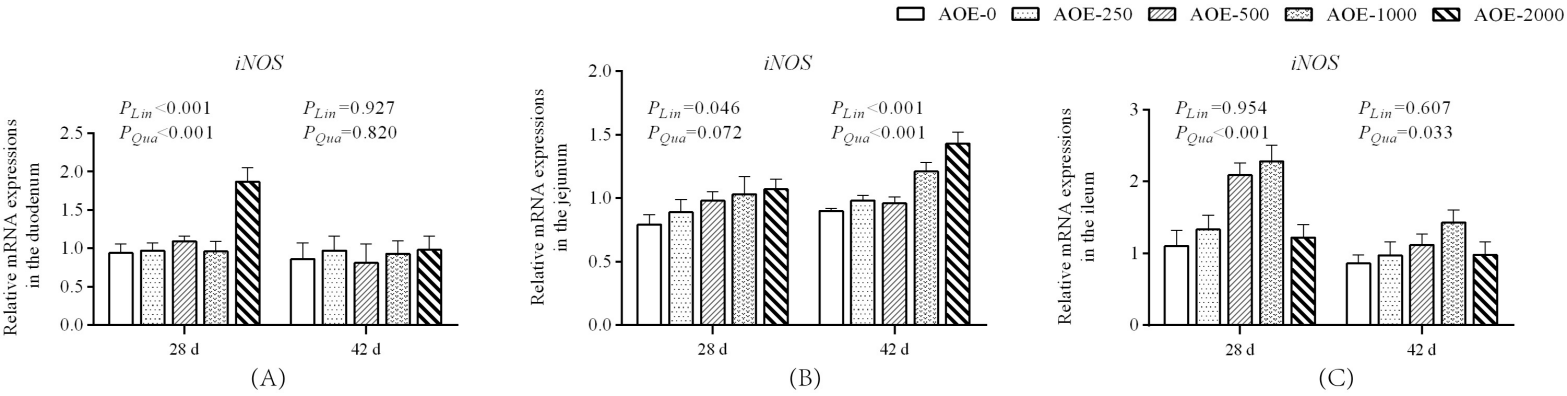
Effects of dietary AOE supplementation on intestinal iNOS mRNA expression in broilers. **(A)** Relative mRNA expression of iNOS in duodenum, **(B)** Relative mRNA expression of iNOS in jejunum, **(C)** Relative mRNA expression of iNOS in ileum. iNOS: Inducible nitric oxide synthase AOE: *Artemisia ordosica Krasch*. aqueous extract. CON: control; AOE-250: 250 mg/kg AOE; AOE-500: 500 mg/kg AOE; AOE-1000: 1,000 mg/kg AOE; AOE-1500: 1,500 mg/kg AOE; AOE-2000: 2,000 mg/kg AOE.

The effects of dietary AOE supplementation on intestinal *iNOS* mRNA expression in broilers are presented in Figure 4. In the duodenum, as dietary AOE levels increased, *iNOS* mRNA expression increased linearly and quadratically at 28 days of age (*P* < 0.001; *P* < 0.001), with no significant effect at 42 days (*P* > 0.05). In the jejunum, *iNOS* mRNA expression increased linearly at 28 days of age (*P* = 0.046), which exhibited significantly linear or quadratic increase at 42 days of age with increasing AOE levels (*P* < 0.001; *P* < 0.001). In the ileum, as dietary AOE levels increased, *iNOS* mRNA expression exhibited significantly quadratic increase at 28 days of age (*P* < 0.001), which exhibited significant quadratic increase at 42 days of age (*P* = 0.033).

## 4 Discussion

*Artemisia ordosica Krasch*. and its extracts are abundant in bioactive compounds including polysaccharides, flavonoids, organic acids and terpenoids, which can promote growth performance in animals. Previous study by our research team have confirmed that dietary AOE supplementation significantly increased average daily gain, with no remarkable alterations in feed intake and feed-to-gain ratio (25), suggesting the potential application of AOE for enhancing productive performance in broilers. The improvement of growth performance by AOE is closely associated with intestinal immunity and antioxidant function. To elucidate the underlying mechanisms, this study investigated serum and intestinal tissue antioxidant and immune indexes in broilers fed with AOE. In the present study, we found that dietary AOE supplementation effectively enhanced the activity of key antioxidant enzymes and reduced lipid peroxidation products in both serum and intestinal tissues. Meanwhile, it modulated the levels of serum cytokines and improved Newcastle disease antibody titers, accompanied by altered mRNA expression of antioxidant related genes and iNOS in intestinal segments. In terms of mechanism, the antioxidant enhancement by AOE is associated with the up-regulation of intestinal CAT, SOD, and GSH-Px mRNA expression, which provides a molecular basis for the increased antioxidant enzyme activity. Moreover, its immunomodulatory effects are linked to the regulation of serum cytokine balance and the induction of intestinal iNOS mRNA expression. For antioxidant regulation, accumulating evidence has demonstrated that plant-derived extracts rich in flavonoids and polysaccharides typically exert antioxidant effects by modulating the NRF2/KEAP1 signaling pathway, this process could potentially enhance the transcription of antioxidant enzyme genes (e.g., CAT, SOD, GSH-Px) by facilitating NRF2 binding to AREs in their promoter regions (26–28). In our study, dietary supplementation with AOE upregulated the mRNA expression of antioxidant genes (CAT, SOD and GSH-Px) in the duodenum and ileum. It is reasonable to infer that bioactive component in AOE, such as flavonoids, polysaccharides, and phenolic acids, may promote the synthesis of antioxidant enzymes through NRF2/KEAP1 pathway, which synergistically scavenge ROS through sequential reactions—SOD mediating the dismutation of superoxide anions and CAT/GSH-Px decomposing hydrogen peroxide—ultimately alleviating lipid peroxidation and enhancing systemic and intestinal antioxidant capacity (29, 30). In terms of immune modulation, AOE's regulatory effects are presumably mediated through the TLR4/NF-κB-iNOS-NO signaling cascade, a classical pathway widely implicated in plant-derived extract-induced immune activation in poultry (31–35). Previous studies have confirmed that bioactive components (e.g., polysaccharides, flavonoids) from medicinal plants can bind to TLR4 on immune cell membranes. This binding initiates downstream signaling events that promote IκB phosphorylation and NF-κB nuclear translocation—ultimately inducing the transcription of immune-related genes such as iNOS and pro-inflammatory cytokines (36). Similarly, dietary supplementation with AOE also promoted the mRNA expression of iNOS in the intestine tissues, which was accompanied by the increase of serum NO content and iNOS activity in our study. Previous research has demonstrated that the moderate increase in NO production, driven by iNOS activity upregulation, plays a dual immunomodulatory role. It enhances host defense by promoting macrophage phagocytosis, T/B lymphocyte proliferation and differentiation, and specific antibody production, while avoiding excessive cytotoxicity (37).

Numerous studies have confirmed that plant-derived extracts rich in flavonoids and polysaccharides can synergistically enhance poultry antioxidant and immune functions by regulating endogenous molecular targets (38, 39). For instance, *A. ordosica* extract (flavonoids, phenolics, and terpenoids) can alleviate the colitis induced by dextran sulfate sodium in mice by regulating inflammatory responses, repairing the intestinal barrier, and enhancing the activity of intestinal antioxidant enzymes (40). Similarly, *A. ordosica* polysaccharides have been shown to effectively alleviate lipopolysaccharide-induced oxidative stress injury in poultry peripheral blood lymphocytes by activating the NRF2/KEAP1 pathway and regulating the TLR4/NF-κB pathway (41). Consistent with these reports, our study demonstrated that dietary AOE supplementation alleviated oxidative stress and optimized immune responses in broilers. Notably, the regulatory effects of AOE exhibited a clear dose-dependent pattern: the 1,000 mg/kg supplementation level showed relatively pronounced effects in enhancing antioxidant enzyme activity, regulating cytokine balance, and upregulating the expression of target genes (antioxidant genes, iNOS). In contrast, the 2,000 mg/kg dose failed to further improve the above regulatory efficacy and even exerted a mild negative effect. The dose-related pattern of AOE's efficacy may reflect the threshold dependent activation of its target signaling pathways. Specifically, low doses may not provide sufficient bioactive components to trigger effective pathway activation, while excessive doses might lead to competitive inhibition or negative feedback regulation at the molecular level, thereby limiting further enhancement of regulatory effects (17, 42). A notable finding of this study is that the water-extracted AOE exhibits distinct practical advantages compared with the previously reported A. ordosica extracts and isolated polysaccharides. Firstly, water extraction is a green and safe preparation method that avoids organic solvent residues, thereby better aligning with the safety requirements of feed additives for broiler production. Secondly, water extraction may effectively retain heat-stable bioactive components with high bioavailability from A. ordosica (flavonoids, polysaccharides, phenolic acids) (22). Thirdly, water extraction features simple operation and low production cost, which is more suitable for largescale industrial application in broiler farming. However, this study faces certain initial challenges: the specific content and composition of bioactive components in AOE have not been determined, and the key active ingredients mediating its antioxidant and immune regulatory effects remain unclear.

Notably, in this study both the antioxidant and immunomodulatory effects of AOE exhibit consistent segment-specific and age-dependent characteristics, which can be explained by the inherent physiological traits of broilers and the functional heterogeneity of intestinal segments. Regarding intestinal segment differences, the ileum—a core mucosal immune site enriched with immune cells (e.g., macrophages, lymphocytes) in poultry (1)—exhibits robust transcriptional responses of both antioxidant genes and the immune-related *iNOS* gene. This aligns with its function as immune cell activation generates substantial ROS needing scavenging by antioxidant enzymes and requires iNOS-induced NO to enhance immune function. In contrast, the duodenum, focused on nutrient digestion, has sparse immune cells and ROS primarily from metabolism, leading to only mild early upregulation of CAT/SOD and minimal iNOS induction. The jejunum, a transitional segment specialized for nutrient transport, shows weak responses in both antioxidant and *iNOS* gene expression due to low oxidative stress and limited immune involvement, aligning with its functional distinction from the ileum (43–45). For age-dependent variations, it is important to consider that the antioxidant and immune systems of broilers gradually mature with growth: early in the trial (15~28 days of age), transcriptional activation of key genes lays a foundation for subsequent functional enhancement, while in the late growth phase (29~42 days of age), elevated metabolic rates and cumulative environmental stress increase demands for antioxidant and immune defense, allowing AOE's regulatory effects to be more fully manifested (44). Furthermore, the antioxidant and immunomodulatory effects of AOE are not mutually exclusive but form a synergistic regulatory network. Enhanced antioxidant capacity helps maintain redox homeostasis in immune cells, preventing ROS-induced damage to their proliferation and secretory functions, which in turn facilitates the efficient activation of immune-related signaling pathways such as TLR4/NF-κB (46). Conversely, moderately increased NO production may inhibit NADPH oxidase activity to reduce ROS generation (33), complementing the ROS-scavenging effects of antioxidant enzymes. This crosstalk between antioxidant defense and immune activation highlights the comprehensive regulatory potential of AOE, which integrates multiple bioactive components to target interconnected molecular pathways (47).

Despite the valuable insights gained, this study has certain limitations that warrant further investigation. Firstly, while we inferred the involvement of NRF2/KEAP1 and TLR4/NF-κB-iNOS-NO pathways based on mRNA expression and existing literature, this study only detected the transcriptional level of target genes, without verifying the protein expression and activation status of key pathway molecules (e.g., *Nrf2* nuclear translocation, *NF-*κ*B* phosphorylation, *iNOS* protein levels). This limits the direct confirmation of pathway activation and the precise mechanistic link between AOE and its regulatory effects. Secondly, the specific content and composition of bioactive components in AOE (e.g., individual flavonoids, polysaccharide subtypes) remain undetermined, making it difficult to identify the key ingredients mediating the observed antioxidant and immunomodulatory effects. Future research will focus on the qualitative and quantitative analysis of bioactive components in AOE, as well as verifying the protein expression and activation of core pathway molecules. These efforts will further fortify the theoretical foundation for the rational application of AOE as a green feed additive in broiler production.

## 5 Conclusions

In conclusion, dietary supplementation with AOE exerted positive regulatory effects on the antioxidant capacity and immune function of broilers. Specifically, AOE improved the antioxidant status of broilers by increased serum CAT activity and reduced MDA concentration at 42 days of age. Concurrently, it enhanced intestinal antioxidant defenses by increasing the activity of GSH-Px, SOD, CAT and T-AOC in the duodenum, jejunum and ileum, and decreasing ileal MDA content. Regarding immune modulation, AOE elevated the levels of serum cytokines (IL-2, IL-1β, IL-6 and TNF-α) at 28 days of age and boosted the Newcastle disease antibody titers at both 28 and 42 days of age, thereby improving the immune response ability of broilers. At the molecular level, AOE upregulated the mRNA expression of antioxidant genes (*CAT, SOD* and *GSH-Px*) in the duodenum and ileum at 28 days of age. It also promoted the mRNA expression of *iNOS* in the intestine, which was accompanied by the increased of serum NO content and iNOS activity. The dose-response analysis showed that the regulatory effect of AOE on broilers is most appropriate when the dietary supplementation level was 1,000 mg/kg, and excessive supplementation (2,000 mg/kg) did not confer additional benefits. Overall, this study confirms that AOE is a promising natural green feed additive, which can effectively improve the health status of broilers by enhancing their antioxidant and immune functions, and provide a scientific basis for the rational application of AOE in broiler production.

## Data Availability

The original contributions presented in the study are included in the article/supplementary material, further inquiries can be directed to the corresponding author.
